# Transoral endoscopic parathyroidectomy vestibular approach: our experience from a single center in Kuwait

**DOI:** 10.1097/MS9.0000000000003831

**Published:** 2025-09-04

**Authors:** Jana Mohammad, Dalia Albloushi, Sood AlSairefi, Lubna Tofesh, Abeer AlSubaiei, Salman Alsafran

**Affiliations:** a Department of Surgery, Jaber Al-Ahmed Hospital, Kuwait; b Department of Surgery, Mubarak Al-Kabeer Hospital, Kuwait; c Department of Surgery, Kuwait University, Kuwait

**Keywords:** case series, endocrine surgery, parathyroidectomy, transoral vestibular approach

## Abstract

**Objective and background:**

We present our experience with the first five parathyroidectomies performed in Kuwait using the transoral endoscopic vestibular approach.

**Methods:**

Retrospective data collection of trans-oral endoscopic vestibular parathyroidectomies was performed at a single institution in Kuwait. Patient eligibility was determined by previously published exclusion criteria. Information on patient demographics, perioperative management, and complications was collected and reviewed.

**Outcomes:**

In our case series, we present five patients who underwent transoral endoscopic parathyroidectomy via the vestibular approach. All included patients had a biochemical diagnosis of primary hyperparathyroidism and two concordant localizing studies demonstrated a single parathyroid adenoma. All patients were above 18 years of age, had no previous neck surgery, and were not on anticoagulants. No intraoperative complications were reported, and the mean operative time was 180 minutes. Postoperatively, four patients were discharged on the same day while one patient was discharged on postoperative day 2. During follow-up, no complications were reported, and the patients reported satisfactory cosmetic results. Finally, parathyroid adenoma was confirmed in the final histopathology report in all five cases.

**Conclusion:**

The transoral endoscopic parathyroidectomy vestibular approach (TOEPVA) is a viable, safe, and cosmetically advantageous option for parathyroidectomy in select patients. Careful patient selection is crucial, with criteria including a single adenoma identified on preoperative imaging, and no history of neck surgery or anticoagulant use. Further research is needed to clarify TOEPVA’s role in the surgical management of primary hyperparathyroidism and to explore its long-term outcomes in a larger, diverse patient population.

## Introduction

Hyperparathyroidism was first defined in the 1920s by Albright and Recklinghausen in the context of severe bone disease^[1]^. A single benign adenoma is the cause of primary hyperparathyroidism in 80–85% of cases, while multiple adenomas contributes to 10–15% of cases, and parathyroid carcinoma causes less than 1% of cases^[1]^. Patients with hyperparathyroidism present with high levels of serum calcium resulting from excessive production of parathyroid hormone (PTH)^[1]^.

Surgical excision is the gold standard treatment for primary hyperthyroidism, with an effective cure rate of 96.5%^[2]^. There are multiple surgical techniques to achieve parathyroidectomy, including open bilateral or unilateral cervical exploration, minimally invasive open surgery guided by gammagraphy, video-aided minimally invasive surgery, and endoscopic surgery via the axilla^[3]^. Recently, the transoral endoscopic parathyroidectomy vestibular approach (TOEPVA) has been described as a feasible and successful alternative for surgical treatment of parathyroid disease. This novel technique was first illustrated by Karakas *et al*^[4]^ in 2010, with no documented intraoperative complications.

**HIGHLIGHTS**

- This is the first case series in Kuwait evaluating the transoral endoscopic parathyroidectomy vestibular approach (TOEPVA).
- All five patients achieved successful biochemical cure without intraoperative or postoperative complications.
- TOEPVA provided a scarless surgical option with high patient satisfaction regarding cosmetic outcomes.
- Strict patient selection was essential, with inclusion criteria based on preoperative localization and absence of prior neck surgery.
- Findings support TOEPVA as a feasible, safe, and cosmetically superior alternative for select patients with primary hyperparathyroidism.

The foremost advantage of TOEPVA is the cosmesis results. However, various remote-access surgical techniques have been developed to reduce visible scarring, including transaxillary approaches or endoscopic total parathyroidectomy via anterior chest approach. Both approaches have been associated with specific downfalls, for instance, the transaxillary approach provides adequate lateral access; however, it is associated with a wider dissection field and operative time – increasing the patients morbidity^[5,6]^. Furthermore, the endoscopic total parathyroidectomy via anterior chest approach offers a more central view, though it is associated with a high risk of seroma formation and is more technically demanding^[7]^. In contrast, TOEPVA offers a midline direct access to the parathyroid glands with limited dissection and no external scars^[8,9]^. It has been reported that TOEPVA accomplishes comparable results to the traditional open parathyroidectomy when performed by experiences surgeons, with a learning curve of 7–15 cases^[10,11]^ and is associated with higher cosmetic satisfaction, particularly in those prone to hypertrophic scarring^[12,13]^. While widely popular in East Asia and increasing popularity in North America and Europe, TOEPVA remains underutilized in the Middle East.

This case series, the first documented report from the Middle East, presents our experience with TOEPVA from a single center in Kuwait.

## Materials and methods

### Study population and design

We conducted a retrospective case series of all TOEPVA procedures performed between 2019 and 2025 at a single governmental institution by a general surgeon with fellowship training in endocrine surgery, who additionally trained under Dr. Angkoon Anuwong in Thailand. The work has been submitted with a research registry; unique identifying number is research registry number 11093. The following case series is in compliance with the PROCESS guideline^[14]^.

Russel *et al* – a 2020 update, proposed that surgical candidates for TOEPVA should have localized primary hyperparathyroidism. Contraindications included multi-gland disease, recurrent or persistent hyperparathyroidism, family history of multiple endocrine neoplasia (MEN), suspected carcinoma, secondary or tertiary hyperparathyroidism, and previous neck irradiation or surgery. The relative contraindications include smoking and morbid obesity^[5]^.

### Perioperative course

All included patients had a biochemical diagnosis of primary hyperparathyroidism and two concordant localizing studies demonstrating a single parathyroid adenoma, namely, ultrasound, sestamibi scan, and 4D computed tomography. The operating surgeon then confirmed the findings using a bedside ultrasonography prior to the incision. All the patients underwent preoperative vocal cord assessment, which revealed no abnormalities. The patients were kept nil by mouth prior to surgery, and a single dose of intravenous cefazolin was administered. The surgical approach adopted was based on the technique described by Sasanakietkul *et al* for TOEPVA^[8]^.

### Intraoperative course

**Figure 1.**
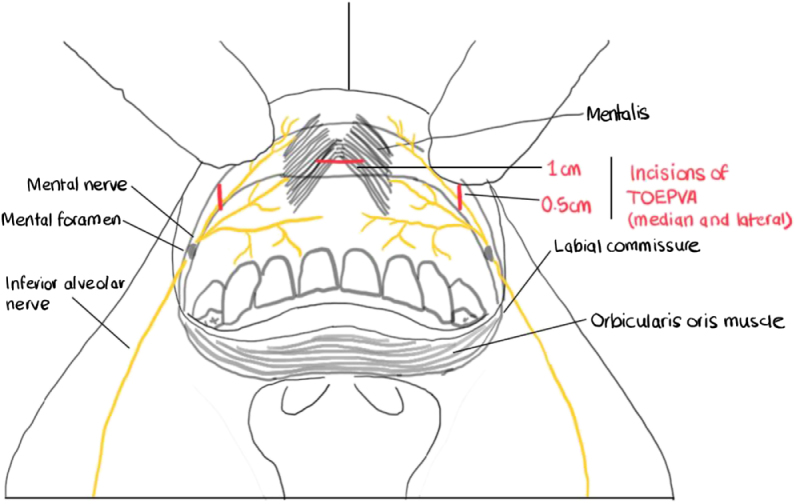
Schematic illustration of the TOEPVA access route and port placement. TOEPVA, transoral endoscopic parathyroidectomy vestibular approach.

The procedure began with a 10 mm horizontal incision in the oral vestibule above the buccal frenulum, extending through the mucosa and muscular layer. A submuscular flap was created caudally to the mental protuberance using electrocautery, followed by tumescent dissection with a Veress needle and epinephrine solution in normal saline to develop a subplatysmal space extending from the mandible to the clavicle. A 10 mm trocar was placed in the central incision for insufflation to 7 mmHg, and an additional 5 mm ports was created laterally at the vermillion border near the canine and first premolar junctions bilaterally (Fig. 1).

**Figure 2.**
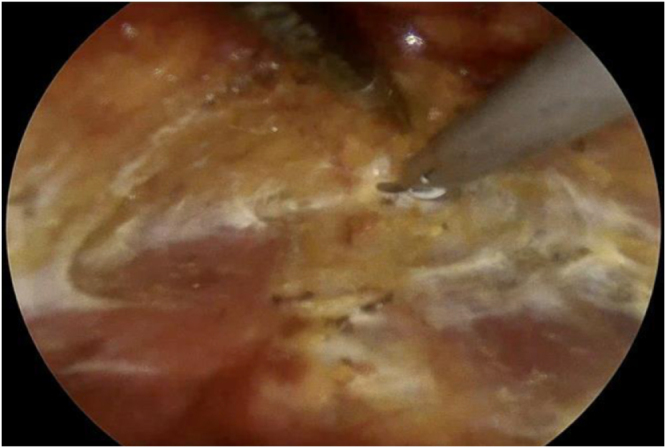
Dissection of the subplatysmal plane.

**Figure 3.**
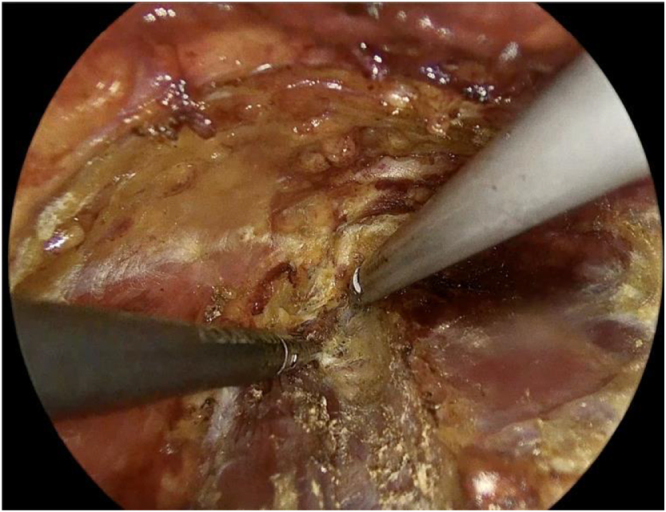
The division of the strap muscles.

A 30-degree endoscope was introduced through the 10 mm port, with Maryland dissectors and hook cautery used to dissect the subplatysmal plane (Fig. 2) and to open the midline between the strap muscles (Fig. 3).

**Figure 4.**
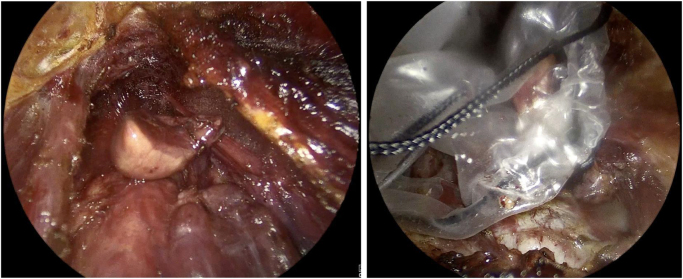
Identification and dissection of the target parathyroid adenoma.

A LigaSure device facilitated dissection and hemostasis, allowing for bilateral exposure of the strap muscles, the thyroid, and the parathyroid glands. The recurrent laryngeal nerve (RLN) was identified using an NIM (Nerve Integrity Monitor) probe to ensure its preservation. The target parathyroid gland was excised with the LigaSure device and retrieved through the central 10 mm port using a specimen bag (Fig. 4).

### Closure and recovery

**Figure 5.**
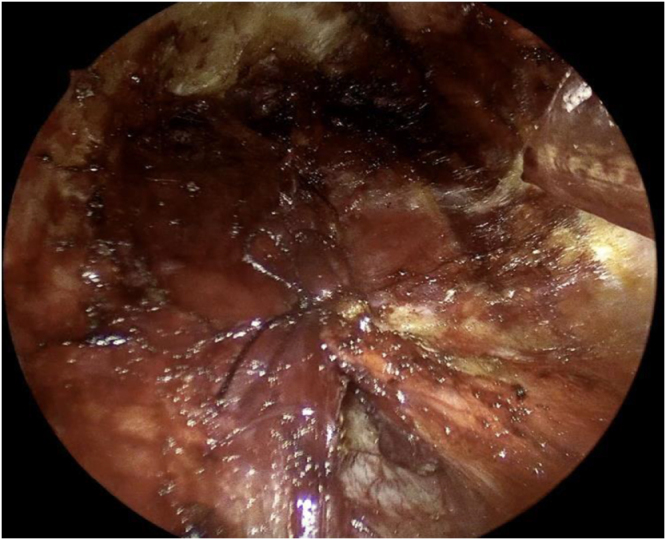
Closure of the strap muscles using 3-0 Vicryl.

The strap muscles were approximated with 3-0 Vicryl sutures (Fig. 5). The lip incisions were closed in two layers (muscular with 3-0 Vicryl and mucosal with 4-0 Vicryl sutures). A jaw bra was applied postoperatively to support the neck and jaw and prevent postoperative hematoma. The patient tolerated the procedure well, was extubated without complications, and was transferred to recovery.

This technique highlights the scarless transoral approach with meticulous dissection, RLN preservation, and effective hemostasis, aligning with published protocols that emphasize patient safety, cosmetic outcomes, and surgical efficacy.

### Postoperative course

Immediately after surgery, the patients underwent vocal cord examination with a laryngoscope to assess RLN function. After the cessation of the anesthetic effect, patients resumed oral intake and were instructed to avoid pulling on their lower lip. A Jaw bra, which is a pressure dressing, was advised for 7 days. The discharge criteria included effective pain management with oral analgesics, the ability to tolerate oral intake without nausea or vomiting, and no immediate postoperative complications.

## Results

In our case series, we present five patients who underwent transoral endoscopic parathyroidectomy via the vestibular approach.

Patient characteristics are given in Table 1.

**Table 1:** Patient characteristics

Case no.	Age	Gender	Comorbidities	Laterality	Preoperative imaging
1	50	Female	Nil	Right inferior	0.4 × 0.5 × 0.9 cm
2	22	Male	DM	Left inferior	1.7 × 1.6 × 2.2 cm
3	47	Female	IDA	Right inferior	1.8 × 1.6 × 0.7 cm
4	61	Female	Osteoporosis	Left superior	1.9 × 1.4 cm
5	46	Female	Nil	Right superior	1.6 × 0.5 cm

Four out of the five cases were female. Their ages ranged from 22 to 61 years old, mean age of 41.5. The maximum diameter of the parathyroid gland is 2.2 cm. All included patients had a biochemical diagnosis of primary hyperparathyroidism, and two concordant localizing studies demonstrated a single parathyroid adenoma. However, during surgery, one patient was found to have a superior adenoma instead of an inferior adenoma, as indicated by imaging. All patients were above 18 years of age, had no previous neck surgery, and were not on anticoagulants.

Operative details are summarized in Table 2.

**Table 2:** Operative details

Case no.	Extent of surgery	Operative time (minutes)	Estimated blood loss	Length of hospital stay (hours)	Final pathology	Postoperative complications
1	Right inferior	205	50cc	24	Right inferior parathyroid adenoma	Nil.
2	Left superior	200	Nil.	24	Left superior parathyroid adenoma	Nil.
3	Right inferior	90	Nil.	24	Right inferior parathyroid adenoma	Nil.
4	Left superior	225	5cc	48	Left superior parathyroid adenoma	Nil.
5	Right superior	110	Nil.	24	Right superior parathyroid adenoma	Nil.

There were no reported intraoperative complications, with a mean operative time of 180 minutes, range: 90–225 minutes. Intraoperative PTH monitoring was performed using the Miami criteria, with four blood samples collected at key time points: pre-incision, post-incision, 5 minutes post-removal, and 10 minutes post-removal. This confirmed the successful removal of the adenoma. The PTH monitoring process took 45–60 minutes, which may account for the prolonged operative time.

Postoperatively, four patients were discharged on the same day, while one patient remained hospitalized until postoperative day 2. Six-month follow-up lab results showed no evidence of persistent disease.

Throughout follow-up, no complications were reported, including voice changes, hypocalcemia, or mental nerve injury. Patients also expressed satisfaction with their cosmetic outcomes. Finally, parathyroid adenoma was confirmed in the final histopathology report for all five cases.

## Discussion

This case series presents our initial experience with TOEPVA for primary hyperparathyroidism, representing the first documented cases from a single center in Kuwait. All five patients in this series achieved successful outcomes without intraoperative complications, with no conversions to open surgery. Cosmetic satisfaction was high among all patients, with no visible scars, and all achieved biochemical remission postoperatively. This reinforces TOEPVA as a feasible and effective alternative to traditional parathyroidectomy techniques, particularly in patients who prioritize a scarless approach.

### Comparison to traditional approaches

Historically, parathyroidectomy has been performed via the trans-cervical route, which is associated with excellent cure rates, but leaves a permanent anterior neck scar. Several studies have highlighted the psychosocial impact of neck scars on patients’ quality of life, particularly in younger individuals and those with a predisposition to hypertrophic scarring^[3]^. Choi *et al*^[12]^ studied the effect of neck scars on the quality of life of 92 patients who underwent a thyroidectomy. Post-thyroidectomy scars seem to adversely affect the quality of life of patient with thyroid cancer, regardless of scar appearance. Others have found that the appearance of a scar is the most commonly reported adverse event^[4,12]^, and that this can affect the postoperative quality of life for some patients more than others.

TOEPVA offers a distinct advantage by eliminating visible scarring, thereby potentially improving quality of life. In our series, all patients reported satisfaction with the cosmetic outcomes, mirroring the results of Kasemsiri *et al* which showed a statistically significant difference in cosmetic outcomes and overall satisfaction when comparing TOEPVA with the traditional approach^[13]^. Another study surveyed post-thyroidectomy patients and found that patients with a traditional neck incision had decreased quality of life that can be comparable to those with chronic skin diseases such as psoriasis and severe atopic eczema^[3]^.

In addition to cosmetic benefits, TOEPVA allows for minimizes tissue dissection and preserves critical structures while offering a favorable operative field for visualizing the recurrent laryngeal nerves bilaterally^[7]^. The reduced tissue manipulation aligns with the observed lower analgesic requirements and shorter hospital stays, with four of five patients discharged within 24 hours in this series. These findings are consistent with the work of Makay *et al*^[6]^, who reported reduced pain scores and shorter hospital stays for TOEPVA patients than for those undergoing open parathyroidectomy.

The transoral approach to the neck is classified as “clean-contaminated” surgery and carries an inherently greater risk of infection than the clean transcervical approach^[5]^. However, several high-volume thyroid surgeons in the United States and Turkey showed similar rates of wound infections when comparing the transoral and transcervical approaches^[7,15]^. In our study, none of the patients reported intra-oral infections postoperatively. Some reported complications limited to the transoral vestibular approach reported include temporary chin numbness from mental nerve injury and oral commissure tears^[10]^; this was not demonstrated in our study^[16]^. Chen *et al* conducted a systematic review of over 860 cases of Transoral Endoscopic Parathyroidectomy Vestibular and found that the transoral vestibular approach could be a safe and feasible approach to thyroid surgery with a 99% success rate^[16]^. Similarly, a systematic review done by Entezami *et al* suggested that the same approach can be used for parathyroid gland removal in a *carefully selected patient cohort*, with a success rate of 96% and a complication rate of 3.8%, comparable to the traditional open approach^[17]^. However, owing to the small sample size and low quality of evidence with risk of bias, larger scale randomized controlled trials are required^[7,17]^.

The mean operative time in our series was 180 minutes, which is longer than other reported series^[6,7,9,10]^, and this can be attributed to intraoperative PTH monitoring protocols that added 45–60 minutes per procedure.

Table 3 shows a comparison of TOEPVA case series in literature.

**Table 3:** Comparison of TOEPVA case series in literature

Study	No. of Patients.	Mean Operative Time (min)	Conversion Rate (%)	Conversion Rate (%)	Biochemical Cure (%)
Current Study	5	180	0	0	100
Russell et al.(2018)^[7]^	10	127	0	0	100
Makay et al. (2022)^[6]^	35	116	19 (ecchymosis, emphysema, nasal bleeding, seroma, infection)	8.6(3/35)	100
Kasemsiri et al (2020)^[9]^	8	125	0	0	100
Grogan et al (2022)^[18]^	101	130.5 →66.5^a^	1(1transient RLN palsy)	0	98
Sasanakietkul et al (2017)^[8]^	12	107.5	8.3(1/12 transient RLN injury)	Not reported	100
Khafif et al (2021)^[19]^	19	NR	10.5%(2/19 mental nerve injuries	0%	100%

^a^ Grogan et al. noted a learning curve, with operative time dropping from 130.5 minutes in the first half to 66.5 minute in the second half

### Limitations of TOEPVA and importance of patient selection

Although TOEPVA shows promise, its adoption should be guided by strict selection criteria to ensure patient safety and optimal outcomes. The inclusion criteria in this series required patients to have a single adenoma identified on preoperative imaging and no history of neck surgery or anticoagulant use. This cautious selection mirrors the recommendations by Razavi *et al* ^[11]^, which emphasize preoperative localization and assessment of factors such as hypertrophic scarring potential and patient motivation to avoid a cervical scar^[11]^. Given the inherent technical limitations of TOEPVA, such as a 2D operative field and limited space for instruments, patients with multiple adenomas, recurrent disease, or prior neck surgeries are not ideal candidates. Grogan *et al*^[20]^ further supported this finding, showing that previous neck surgery was one of the most common reasons for ineligibility for transoral Endocrine surgery (TES), including TOEPVA. In their study, 22% of patients were deemed ineligible due to prior neck operations, highlighting the need for careful patient selection to avoid intraoperative challenges^[20]^15. Additionally, relative contraindications such as smoking or morbid obesity may increase the complexity of TOEPVA. Obesity, in particular, can complicate the elevation of skin flaps, prolonged operative time, and increased the risk of conversion to open surgery^[5,20]^. Grogan *et al* found that the mean body mass index (BMI) of TES-eligible patients was lower than that of ineligible patients, suggesting that a higher BMI may negatively impact candidacy for TOEPVA^[20]^.

As experience with TOEPVA increases, defining and refining patient eligibility criteria will be essential to minimize complications and ensure consistency in outcomes. Patient selection for TOEPVA was reviewed by Ranganath *et al*^[21]^, who suggested that TOEPVA should be performed only in cases of primary hyperparathyroidism after accurate anatomical localization of a parathyroid adenoma via imaging. Additional contraindications – identified in other minimally invasive parathyroidectomy approaches – include suspected multi-gland disease, equivocal localization studies, a family history of MEN, suspected parathyroid carcinoma, and known contralateral RLN injury. These findings align with those of Grogan *et al*^[20]^, who reported that 17.7% of patients were ineligible for TES because of non-localized primary hyperparathyroidism, reinforcing the importance of preoperative imaging and careful patient selection for TOEPVA.

### Technical considerations and challenges

TOEPVA presents several technical advantages, including improved visualization of anatomical structures, such as the recurrent laryngeal nerves and parathyroid glands, which may facilitate the preservation of these critical structures. However, the use of a rigid endoscope with a 2D field can be challenging, particularly for surgeons with no experience in endoscopic procedures. Our findings emphasize the importance of training and familiarity with endoscopic techniques to achieve optimal results. Future initiatives may benefit from standardized training protocols, as suggested by Khafif *et al*, to streamline the learning curve associated with this approach^[19]^.

A study by Razavi and Russell describing the TOETVA learning curve using operative time as a surrogate concluded that 11 cases were needed for procedural proficiency, which is similar to the 15 case curve described for video-assisted thyroidectomy^[11]^. Razavi and Russell found that the operative time began to decrease, without a decrease in success rate or increased complication rate after performing 11 cases^[11]^, which is slightly higher than the 7–10 cases estimated by Anuwong ^[10]^. In addition, Razavi and Russell suggested that the lobe or parathyroid gland targeted for removal should be ipsilateral to the surgeon’s dominant hand during the first surgical procedures^[11]^.

## Implications and future directions

The success of TOEPVA in our case series supports its use as a safe and effective alternative treatment for primary hyperparathyroidism in appropriately selected patients. However, while early outcomes are promising, longer follow-up is necessary to evaluate the durability of biochemical cure rates and to monitor for potential late complications. Additionally, further multicenter studies and randomized controlled trials comparing TOEPVA with traditional parathyroidectomy techniques are needed to provide robust data on long-term efficacy and patient quality of life.

As the TOEPVA approach gains acceptance, future research should focus on refining indications, standardizing preoperative localization protocols, and identifying specific patient populations that may derive the greatest benefit. Given its scarless nature and minimal postoperative discomfort, TOEPVA has significant potential for expanding the scope of minimally invasive endocrine surgery and enhancing patient satisfaction^[13]^.

With the increased use of artificial intelligence in healthcare, we looked into its potential use in TOEPVA. A review study by Apostolopoulos looked at artificial intelligence (AI)’s application in identifying and localizing abnormal parathyroid glands, it demonstrated that deep learning models have shown high diagnostic accuracy when used in tandem with Sestamibi scans and 4D-CT. An AI-driven platform called ParaNet achieved patient-level diagnostic accuracy exceeding 94% and outperformed human interpreters in certain settings.

### Integration of AI in TOEPVA optimization

AI has begun to reshape many aspects of medical research and clinical practice. In molecular biology and drug development, models like AlphaFold have revolutionized the ability to predict protein structures with remarkable accuracy^[22]^. Likewise, image-based AI tools are now being used to support genomic profiling and tailor treatment plans in oncology^[23]^. These innovations suggest that AI may also help address some of the current challenges faced in TOEPVA and improve surgical outcomes.

Currently, TOEPVA relies mainly on conventional imaging, such as ultrasound and sestamibi scans, for preoperative localization. However, these methods can fall short, particularly in cases with multi-gland disease or ectopic parathyroid glands. Emerging deep learning models like nnU-Net offer the potential to improve localization by combining data from multiple imaging sources, including CT, MRI, and ultrasound, into a unified 3D map^[24]^. During surgery, augmented reality (AR) navigation platforms such as ORAR could help overcome the limitations of the two-dimensional operative field by providing real-time, three-dimensional visual guidance, potentially reducing the risk of nerve injury^[25]^.

Postoperative monitoring is another area where AI could make a meaningful impact. Currently, clinical evaluation may fail to detect early signs of complications. AI algorithms that analyze patient vital signs and laboratory results from electronic health records could help predict complications like hypocalcemia after thyroid or parathyroid surgery, offering an earlier window for intervention^[26]^. Together, these advances point to a future where AI is increasingly integrated into the TOEPVA pathway, with the potential to improve surgical precision, patient safety, and overall care quality.

## Conclusion

Our initial experience with TOEPVA demonstrates that it is a safe, effective, and cosmetically superior option for parathyroidectomy in carefully selected patients. All patients in our series achieved biochemical remission without intraoperative complications or conversions to open surgery. These early outcomes highlight the procedure’s potential to minimize surgical morbidity while offering scarless results.

Careful patient selection – specifically, ensuring a single adenoma confirmed on imaging and no prior neck surgery – is essential to maintaining safety and effectiveness. Our findings are consistent with prior studies reporting high patient satisfaction and reduced hospital stays. While these short-term outcomes are encouraging, longer follow-up and larger, multicenter studies are needed to assess long-term efficacy and broader applicability.

As TOEPVA gains traction, future research should aim to refine selection criteria, optimize preoperative localization protocols, and identify which patient groups benefit most. Additionally, the integration of artificial intelligence – including AI-driven imaging, AR navigation, and postoperative risk prediction –represents an exciting avenue to further improve precision, safety, and outcomes in TOEPVA. Given its minimal invasiveness and excellent cosmetic results, TOEPVA holds promise for expanding the landscape of endocrine surgery and enhancing patient-centered care.

## Data Availability

Data sharing is not applicable to this article.

## Research registration unique identifying number (UIN)

Name of the registry: Research registry. Unique Identifying number or registration ID: researchregistry. Hyperlink to your specific registration (must be publicly accessible and will be checked):19.

## Presentation

poster in AACE (American Academy of Clinical Endocrinology).
